# O.3.1-5 Peri-urban landscape as an environment for health enhancing physical activity

**DOI:** 10.1093/eurpub/ckad133.142

**Published:** 2023-09-11

**Authors:** Vita Žlender

**Affiliations:** The Urban planning Institute of the Republic of Slovenia (UIRS), Slovenia; vita.zlender@uirs.si

## Abstract

**Purpose:**

Peri-urban landscape is situated between the rural and the urban and encompasses diverse set of green space types, e.g. large semi-natural areas, woodlands, hills, country parks. Such spaces, suitable for nature recreation, can be rarely found in cities. Accordingly, peri-urban landscape is increasingly important for enabling population to become more physically active. However, previous research has shown that many cities struggle with divergences in the use of these spaces. While some of peri-urban green spaces are under high demand for outdoor recreation, which puts considerable pressure on the landscape and threatens its provisioning of landscape qualities such as tranquillity or biodiversity, others are underused despite their recognised potential for recreation. So far, little attention has been paid to this issue in planning and decision making.

This study first assessed the extent to which peri-urban landscape provides possibility for outdoor recreation. Secondly, it explored how to provide support for spatial planners and local governments in the planning and management of peri-urban green spaces for promotion of outdoor recreation.

**Methods:**

We conducted document analysis, literature review and stakeholders’ interviews in three case study cities in Slovenia: Ljubljana, Koper and Kranj. In the first step, we highlighted any formal recognition of peri-urban landscape use for outdoor recreation and identified spatial actions and their influence on the peri-urban land use. Then we carried out the evaluation of the level of importance given to peri-urban green spaces’ suitability for outdoor recreation by the stakeholders. The data was compiled and compared between the three cities.

**Results:**

We identified policy gaps in all case studies, which pointed to the weak role of spatial planning in protection and management of peri-urban green spaces for outdoor recreation. We also identified the lack of methodologies, tools and inclusion of new approaches, such as ecosystem services framework or nature-based solutions, in the spatial planning and policy making.

**Conclusion:**

Our findings provide a supporting material for local decision makers and spatial planners in their efforts to develop peri-urban green spaces as spaces for promoting health among all demographic groups of local population.

**Funding:**

Slovenian Research Agency (grant numbers V5-2232 and Z5-4589).

